# Injury Risk Predictions in Lunar Terrain Vehicle (LTV) Extravehicular Activities (EVAs): A Pilot Study

**DOI:** 10.1007/s10439-024-03543-8

**Published:** 2024-06-05

**Authors:** Luis Poveda, Karan Devane, Mitesh Lalwala, F. Scott Gayzik, Joel D. Stitzel, Ashley A. Weaver

**Affiliations:** 1 Department of Biomedical Engineering, Center for Injury Biomechanics, Wake Forest University School of Medicine, Winston-Salem, NC 27101 USA; 2 Virginia Tech-Wake Forest Center for Injury Biomechanics, 575 N. Patterson Ave, Suite 530, Winston-Salem, NC 27101 USA

**Keywords:** Spaceflight, Rover, Lunar, Finite element modeling, GHBMC, Biomechanics

## Abstract

Extravehicular activities will play a crucial role in lunar exploration on upcoming Artemis missions and may involve astronauts operating a lunar terrain vehicle (LTV) in a standing posture. This study assessed kinematic response and injury risks using an active muscle human body model (HBM) restrained in an upright posture on the LTV by simulating dynamic acceleration pulses related to lunar surface irregularities. Linear accelerations and rotational displacements of 5 lunar obstacles (3 craters; 2 rocks) over 5 slope inclinations were applied across 25 simulations. All body injury metrics were below NASA’s injury tolerance limits, but compressive forces were highest in the lumbar (250–550N lumbar, tolerance: 5300N) and lower extremity (190–700N tibia, tolerance: 1350N) regions. There was a strong association between the magnitudes of body injury metrics and LTV resultant linear acceleration (*ρ* = 0.70–0.81). There was substantial upper body motion, with maximum forward excursion reaching 375 mm for the head and 260 mm for the chest. Our findings suggest driving a lunar rover in an upright posture for these scenarios is a low severity impact presenting low body injury risks. Injury metrics increased along the load path, from the lower body (highest metrics) to the upper body (lowest metrics). While upper body injury metrics were low, increased body motion could potentially pose a risk of injury from flail and occupant interaction with the surrounding vehicle, suit, and restraint hardware.

**Supplementary Information:**

The online version contains supplementary material available at 10.1007/s10439-024-03543-8.

## Introduction

As the United States prepares for upcoming Artemis missions to send astronauts back to lunar soil [1], the performance of extravehicular activities (EVAs) shortly after landing is expected. To expand future crew excursions and exploration capabilities, a Lunar Terrain Vehicle (LTV) will likely be employed to transport astronauts across the lunar terrain [2]. As part of optimizing vehicle egress and ingress time efficiency, the National Aeronautics and Space Administration (NASA) is considering the adoption of a standing upright posture on the LTV [3]. However, this design raises concerns regarding the higher center of gravity for the crewed vehicle, potentially elevating the risks of vehicle rollover and astronaut ejection in the event of an impact [3]. Moreover, operating an unpressurized vehicle in a standing posture on the uneven lunar surface, with varying slope angles and exposed to the hazards imposed by lunar environmental and topographical conditions, could compromise crewmember safety when they are subjected to transient accelerations caused by lunar surface irregularities such as rocks and craters. Therefore, it is essential to analyze the challenges and risks associated with this proposed posture and operating conditions to ensure the safety and well-being of crewmembers during lunar missions.

Crewed lunar roving vehicle data from earlier Moon missions, as documented in Apollo 15 rover mobility performance reports, provide insights for predicting the mobility performance of an unpressurized vehicle operating in lunar topography and environmental conditions. Lunar topography information from the report indicates the presence of numerous craters surrounding the Apollo 15 landing site, although these craters were characterized by smooth interiors [4]. In addition, a review of post-mission slope distribution estimates from Apollo 15 revealed that slopes at least ± 10° were encountered with > 95% of the total rover EVA traversed distance falling within ± 5° [4]﻿. These estimates and assessments, however, are limited to the Hadley-Apennine (Smooth Mare surface) region of the Moon.

Numerous studies have investigated the effects of transient accelerations on space vehicles and the associated risks of astronaut injuries, employing finite element (FE) modeling techniques during launch, landing, or abort scenarios [5, 6]. However, limited research data are available on transient accelerations experienced by Moon rovers due to lunar surface irregularities. Some studies have examined the vibrational contact performance between a rigid rover wheel and deformable lunar terrain. Although some of these studies do not account for lunar gravity, they reveal that the vertical vibrational acceleration of the rover’s wheel is dependent of the damping effect of the lunar terrain. The majority of vibrational accelerations observed in these studies remained below 5 m/s^2^ at a rover velocity of 10 km/h [7]. Additionally, similar investigations reported maximum rover centroid accelerations magnitudes of 0.5 m/s^2^, 1.5 m/s^2^, and 1.8 m/s^2^ while traversing smooth mare, rough mare, and rough upland terrains, respectively, at a speed of 200 m/h [8].

To investigate the transmission of acceleration pulses to humans within a vehicle, mass transport studies offer insights into the acceleration experienced by standing passengers, serving as approximate estimates for this study. These studies indicate that bus accelerations can reach up to 0.32 G surpassing the balance loss threshold for standing passengers set at 0.15 G [9]. More recent studies have reported that the severity of standing passenger injuries can increase if body balance is lost or the vehicle has struck an object [10]. Additionally, Powell et al. reported at least 50% of the passengers described an acceleration of 2 m/s^2^ or more as unacceptable under jerk profiles of at least 0.52 m/s^3^ [11]. Furthermore, a recent study demonstrated, through a series of tests conducted on volunteers standing on a moving platform, that only a braking pulse of magnitude 1 m/s^2^ could effectively maintain body balance without the need for additional steps [12]. Despite numerous mass transport studies characterizing body kinematics and identifying the step strategy as the primary method to preserve balance while standing and encountering perturbations, ongoing research continues to reveal new strategies [13]. Alternatively, NASA has previously defined 2.7 G in the positive direction as the human tolerance threshold for dynamic response, although this value can be as low as 2.0 G when accounting for spaceflight deconditioning [3]. Moreover, predicted injury risk probabilities remained higher in the lower extremities in an urban bus study conducted by Palacio et al. [9]. Considering the unique conditions of lunar gravity and the act of standing in an unpressurized vehicle, the potential hazard of astronaut ejection must be taken into account [5]. Hence, a comprehensive assessment of the restraining systems is necessary, with particular attention to the limited available data on the interaction between the crewmember and the seats or restraints [5]. Studies on mass transport also contribute in understanding the interactions between standing passengers and vehicle components. Prior investigations using computational methods reported that holding a horizontal handrail and facing forward can decrease the risk of injury [14, 15]. An experimental study revealed that ground reaction forces experienced by participants in an upright standing posture, exposed to bus accelerations and decelerations (with pulse magnitudes ranging from − 0.3 to 0.3 g), were higher when standing freely compared to when grasping a strap [16]. In terms of posture comfort during public transportation usage, a study indicated that a ‘sit-standing’ posture is quantitatively more comfortable, as assessed by the Borg scale, than solely standing posture for all organs except the hip [17].

Computational tools play a critical role in quantifying astronaut injury risks. In particular, FE human body models (HBMs) enable the evaluation of body responses to transient accelerations within reduced lunar gravity environments [18]. The aim of this study is to use an active muscle FE HBM to simulate EVA operations with the LTV and assess astronaut kinematics and injury risk when traversing across the lunar surface [19].

## Materials and Methods

### Human Body Model Positioning & LTV Model

**Fig. 1:**
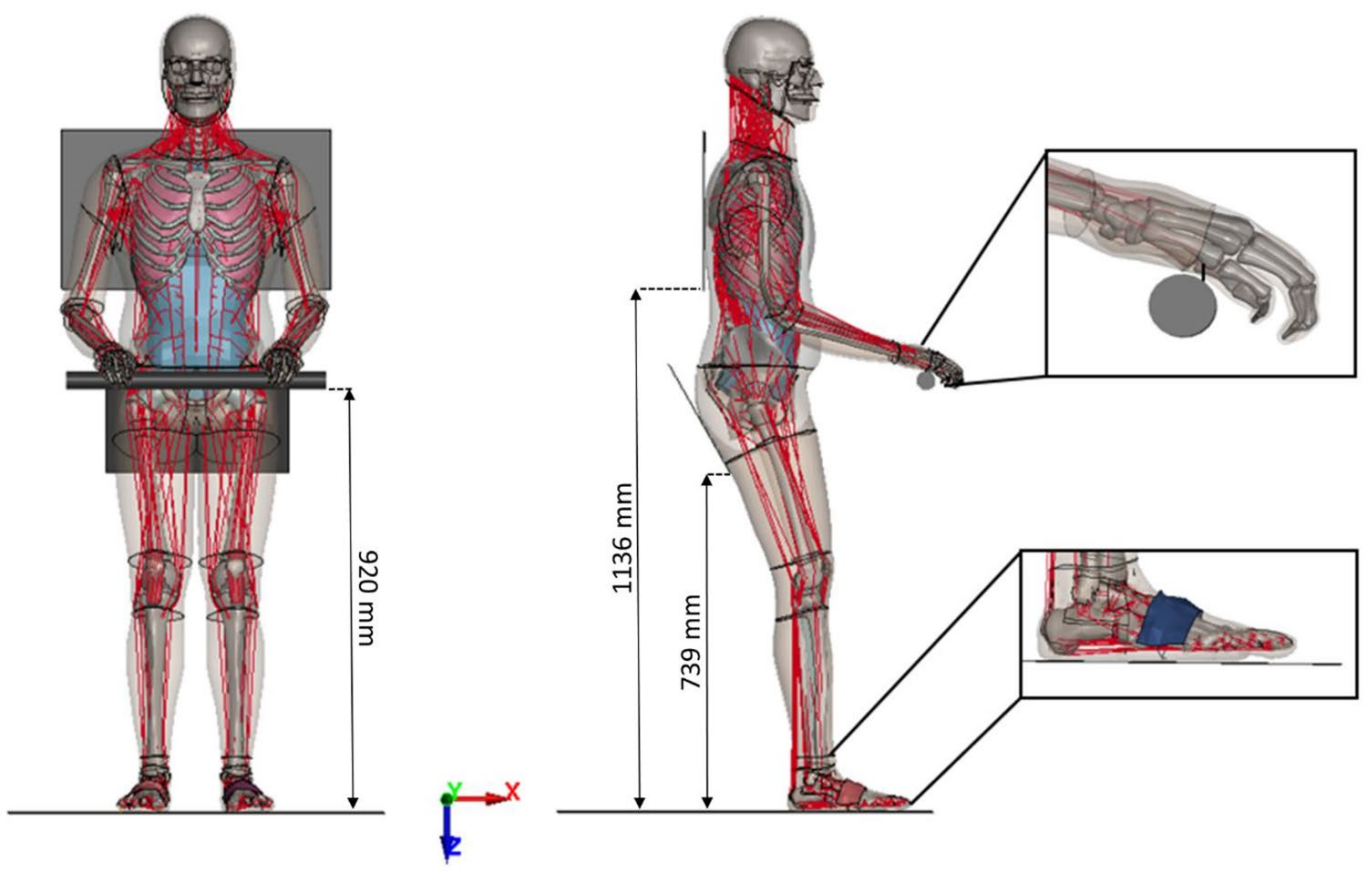
The neutral stance of the GHBMC M50-PS + Active was repositioned to a realistic standing posture on modeled LTV buck. The LTV buck includes a floor panel, seat pan, back rest and handlebar. The inset figures display foot straps and hands tied to the handlebar with beams

The GHBMC 50th percentile male simplified pedestrian model M50-PS + Active [19] (v1.5.2; Elemance, LLC, https://www.elemance.com/models/), initially configured in a neutral standing posture, was repositioned to a semi-standing upright posture, reflecting a realistic posture assumed by astronauts while on the LTV (Fig. 1). This model uses a closed-loop Proportional Integral Derivative (PID) controller strategy for Hill-type beam elements to model muscle activation [19]. Baseline muscle activity used for the muscles was around 0.5%. The chosen crewmember posture draws inspiration from standing desk and scooter ergonomics, as described in prior studies [20–24].

For the LTV seating arrangement, the model was positioned on a rigid seat featuring an inclined pan (388 mm × 268 mm), a rigid vertical backrest (570 mm × 346 mm), and a rigid horizontal footrest (800 mm × 800 mm). Boundary conditions were chosen by drawing inspiration from the Apollo Lunar Roving Vehicle, the conceptual design from a NASA white paper, and considering NASA’s Man-Systems Integration standards (Volume I, Section 3: Anthropometry and Biomechanics) [25, 26]. Material properties were chosen considering the hypothesized small transient acceleration peaks encountered in lunar topography while hitting a crater or rock. Under such conditions, the vehicle buck deformation can be assumed to be negligible for this study. The node spacing on the back rest and rigid seat was no coarser than the outer mesh of the GHBMC model to ensure appropriate distribution of contact forces between deformable and rigid structures. While limited information is available regarding the specific boundary conditions of the actual vehicle, it is anticipated to accommodate two suited astronauts. The hands of the model were connected to a rigid hand gripping console using beam elements with a predefined breaking point of 780 N [9, 27]. Following the repositioning, the model was settled under the influence of gravity (9.81 m/s^2^), ensuring it maintained contact with both the vehicle at the feet, pelvis and back. The final gravity-settled posture was used for all impact simulations as the initial posture. To assess the feasibility of a simplified restraining system, foot straps were incorporated into the model as the primary means of restraint.

### LTV Vehicle Dynamic Data

**Fig. 2:**
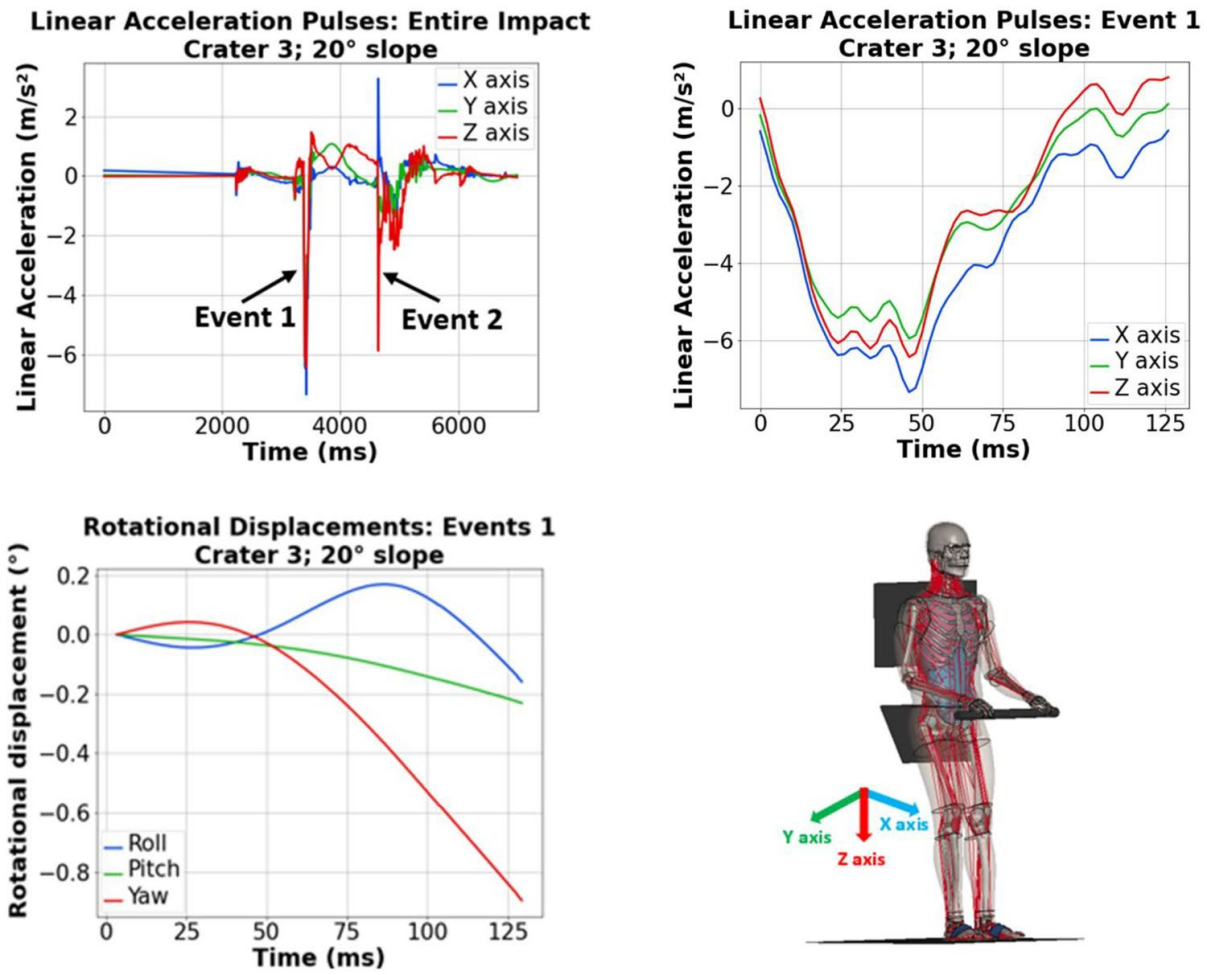
Linear acceleration pulses, with close-up of Event 1, and rotational displacements (roll, pitch, and yaw) for crater 3 (2.0 m diameter & 0.36 m depth) on a 20° slope per the SAE J211 coordinate system [28]

The Simulation and Graphics Branch of the Software, Robotics, and Simulation Division at NASA’s Johnson Space Center conducted a series of simulations of a rover traversing lunar terrain over slope inclinations ranging from 0° to 20° in 5° increments. The right wheels of the modeled rover encountered lunar surface irregularities such as rocks and craters. All simulations were carried out using TRICK (NASA, Houston, TX), an open-source software specifically designed for vehicle development and dynamic load analysis. The resulting vehicle simulation data comprised a total of 25 acceleration profiles for both the right and left seats derived from interactions with 5 different lunar topography irregularities: **crater 1** (1.0 m diameter & 0.15 m depth), **crater 2** (1.8 m diameter & 0.38 m depth), **crater 3** (2.0 m diameter & 0.36 m depth), **rock 1** (10 cm height), and **rock 2** (20 cm height) (Supplementary Material: Fig. 1). Each irregularity was tested across 5 slope inclinations: 0°, 5°, 10°, 15°, and 20°. The acceleration profiles were characterized by tri-axial x-, y-, and z-axis pulses and featured multiple spikes that could be attributed to front and back wheel impacts or the location of wheel impacts on the craters. Notably, two major subsequent spikes were consistently identified across all acceleration pulses. Given that the duration of the impact events in the acceleration signals from the TRICK simulations exceeded typical durations observed in automotive crashes and dynamic spaceflight applications, signal regions surrounding each major spike were truncated and labeled as **event 1** and **event 2**. Figure 2 illustrates the acceleration pulses specifically associated with crater 3 over a 20° slope. On average, these dynamic acceleration pulses had a time duration of 286.2 ± 0.02 ms. In the majority of the cases, the X and Z components of the acceleration pulses had greater magnitudes in comparison to the Y component.

## Finite Element Simulations

To systematically determine which event to simulate for each impact scenario, an algorithm was used to select the most severe event by identifying the highest peak-to-peak values among several vehicle dynamic metrics calculated for each acceleration pulse within a 120 ms time window. This methodology was implemented to ensure the selection of the most severe case was based on the rapid change in acceleration over a short duration and to maintain consistency across all scenarios. The vehicle dynamic metrics considered in the analysis included normalized peak-to-peak resultant acceleration, velocity, and displacement magnitudes, as well as yaw, pitch, and roll rotational angle displacements. The right seat rotational angles of the LTV were derived by calculating the direction cosines of vectors formed from the right seat relative to the left seat. The center of each seat was 766 mm apart. The most severe events for each scenario are summarized in Table 1, which is also the simulation test matrix used. These selected acceleration pulses were utilized to conduct FE simulations in LS-DYNA (version R11.2.2-356; ANSYS, Inc., Livermore, CA) with the GHBMC M50-PS + Active model positioned on the LTV model, accounting for the effects of lunar gravity by applying a gravitational acceleration of 1.63 m/s^2^ in the vertical direction. Acceleration pulses in the *X*, *Y*, and *Z* directions and roll, pitch, and yaw rotational displacements were prescribed as motions in all simulations. A total of 25 simulations were performed, corresponding to the combination of 5 lunar topography impact scenarios (3 craters and 2 rocks) and 5 slope inclinations (0°, 5°, 10°, 15°, and 20°). All simulations were executed on the Distributed Environment for Academic Computing high-performance computing cluster at Wake Forest University (computational time varied from 10 to 18 h per simulation). Each obstacle simulation included a an additional 50 ms lunar gravity-only load after the transient acceleration pulse to prevent the censoring of injury metric and kinematic peak values.

**Table 1:** Test matrix of the 25 modeled LTV scenarios consisting of 3 craters and 2 rocks across 5 different slope inclinations

Slope angles (°)	Crater 1 1.0 m dia. & 0.15 m depth	Crater 2 1.8 m dia. & 0.38 m depth	Crater 3 2.0 m dia. & 0.36 m depth	Rock 1 10 cm height	Rock 2 20 cm height
0	Event 1	Event 1	Event 1	Event 1	Event 1
	5.4 m/s^2^	14.2 m/s^2^	14.1 m/s^2^	7.4 m/s^2^	7.6 m/s^2^
	0.4° yaw	1.0° yaw	1.2° pitch	2.2° pitch	5.0° yaw
5	Event 1	Event 1	Event 1	Event 1	Event 1
	4.9 m/s^2^	14.5 m/s^2^	12.9 m/s^2^	7.2 m/s^2^	7.9 m/s^2^
	0.4° yaw	1.1° pitch	1.° pitch	2.1° pitch	5.3° pitch
10	Event 2	Event 1	Event 1	Event 1	Event 1
	7.14 m/s^2^	10.3 m/s^2^	11.4 m/s^2^	6.9 m/s^2^	7.8 m/s^2^
	0.9° pitch	0.7° yaw	1.0° yaw	2.0° pitch	6.1° pitch
15	Event 2	Event 1	Event 1	Event 1	Event 2
	8.7 m/s^2^	9.6 m/s^2^	12.3 m/s^2^	6.6 m/s^2^	14.9 m/s^2^
	0.9° yaw	0.7° yaw	0.7° yaw	1.8° pitch	6.1° pitch
20	Event 2	Event 2	Event 1	Event 1	Event 2
	7.9 m/s^2^	9.9 m/s^2^	10.8 m/s^2^	6.2 m/s^2^	15.2 m/s^2^
	1.2° yaw	3.1° yaw	1.0° yaw	1.8° pitch	7.2° yaw

Each scenario includes the most severe event along with the corresponding maximum peak-to-peak resultant accelerations and rotational angles

### Data Processing and Injury Metrics

For all simulations, a total of 13 injury metrics were calculated (Table 2). These metrics covered various body regions including the head, neck, lumbar spine and lower extremities. Head kinematics were calculated by extracting nodal accelerations from the center of gravity of the head using the standard instrumentation provided by the GHBMC. Forces and moments acting on the neck were extracted as cross-sectional values at the C2 vertebrae level. Similarly, a cross-section force was obtained for the lumbar spine at the L1 vertebral level. Since this model does not have a deformable spine, a set of deformable elements were added in the middle of the vertebral body, between the rigid elements. The material properties of these elements were taken from the GHBMC M50-PS (v5.3.3; Elemance, LLC).Axial forces were evaluated for the tibia and femur. The peak values were extracted within the dynamic loading time phase. In addition to the injury metrics, body motion envelopes were developed to further understand the kinematic response and excursion of the astronaut’s body. All calculated injury metrics were compared against the Injury Assessment Reference Values (IARVs) based on NASA’s acceptable injury risk tolerance [6].

**Table 2:** Spearman’s *ρ* correlation coefficients for associations between astronaut body injury metrics and LTV dynamic metrics

Spearman’s *ρ* correlation coefficients		LTV resultant linear acceleration		LTV resultant linear velocity		LTV roll angle		LTV pitch angle		LTV Yaw angle	
		*ρ*	*p* value	*ρ*	*p* value	*ρ*	*p* value	*ρ*	*p* value	*ρ*	*p* value
**I**njury metrics	Head linear acceleration	$0.79$	$< 0.0001$	$0.61$	$0.0012$	$0.51$	$0.0094$	$0.38$	$0.0600$	$0.59$	$0.0019$
	Head rotational acceleration	$0.11$	$0.6100$	$0.71$	$< 0.0001$	$0.79$	$< 0.0001$	$0.67$	$0.0003$	$0.68$	$0.0002$
	HIC15	$0.76$	$< 0.0001$	$0.63$	$0.0007$	$0.50$	$0.0100$	$0.39$	$0.0570$	$0.58$	$0.0022$
	BrIC	$0.69$	$0.0002$	$0.54$	$0.0054$	$0.41$	$0.0420$	$0.24$	$0.2600$	$0.41$	$0.0440$
	Peak neck axial force	$0.81$	$< 0.0001$	$0.33$	$0.1100$	$0.15$	$0.4700$	$0.06$	$0.7500$	$0.27$	$0.2000$
	Nij	$0.67$	$0.0003$	$0.52$	$0.0080$	$0.50$	$0.0120$	$0.47$	$0.0200$	$0.63$	$0.0008$
	Peak lumbar axial force	$0.76$	$< 0.0001$	$0.63$	$0.0006$	$0.45$	$0.026$ 0	$0.26$	$0.2140$	$0.46$	$0.0210$
	Peak left femur axial force	$0.76$	$< 0.0001$	$0.51$	$0.0098$	$0.27$	$0.1800$	$0.09$	$0.6800$	$0.31$	$0.1300$
	Peak right femur axial force	$0.56$	$0.0036$	$0.67$	$0.0002$	$0.59$	$0.0019$	$0.45$	$0.0230$	$0.59$	$0.0019$
	Peak left tibia axial force	$0.70$	$< 0.0001$	$0.44$	$0.0300$	$0.22$	$0.2800$	$0.03$	$0.9100$	$0.22$	$0.2900$
	Peak right tibia axial force	$0.70$	$0.0001$	$0.56$	$0.0033$	$0.41$	$0.0420$	$0.21$	$0.3100$	$0.42$	$0.0350$
	Left RTI	$0.73$	$< 0.0001$	$0.65$	$0.0004$	$0.46$	$0.0210$	$0.25$	$0.2100$	$0.46$	$0.0210$
	Right RTI	$0.48$	$0.0143$	$0.82$	$< 0.0001$	$0.74$	$< 0.0001$	$- 0.44$	$0.0023$	$0.70$	$0.0001$

Statistically significant associations are bolded and underlined

### Statistical Analysis

Spearman correlations were computed in JMP Pro 15 (SAS, Cary, NC) to examine the associations between each of the body injury metrics and the LTV dynamic metrics, aiming to identify the most suitable predictors to use in the linear regressions. The Bonferroni correction was applied to account for type I error resulting from the extensive number of tests conducted and the correlation among the 13 injury metrics derived from the same set of 25 simulations. This adjustment yielded a significance level of *α* = (0.05/13 injury metrics*5 LTV dynamic metrics) = (0.05/65) = 0.0008. The resulting Spearman correlation coefficient (*ρ*) and corresponding *p* values were recorded for each test. Spearman correlations were selected to avoid assumptions of normality or linearity, particularly given the small sample size, as it serves as the non-parametric equivalent of the Pearson correlation. To evaluate the predictive capability of LTV linear resultant acceleration for injury metric magnitudes and the proportional variance in injury metrics accounted by LTV linear acceleration, simple linear regression models were developed in R utilizing the non-parametric Kendall-Theil Sen Siegel method, which is a more robust method. Bonferroni correction was used for these tests (*α* = 0.05/13 = 0.004) The R^2^ values were extracted from each regression model, representing the proportion of variance in the injury metric that can be explained by variations in LTV peak-to-peak resultant linear acceleration. An R^2^ of unity signifies that all variations in the corresponding injury metric can be accounted for by variations in linear acceleration. The regression coefficients were also extracted from the regression models, which indicate the change in value of the injury metrics for every m/s^2^ increase in LTV peak-to-peak linear acceleration.

## Results

### Body Kinematics and Injury Metrics

#### Head

**Fig. 3:**
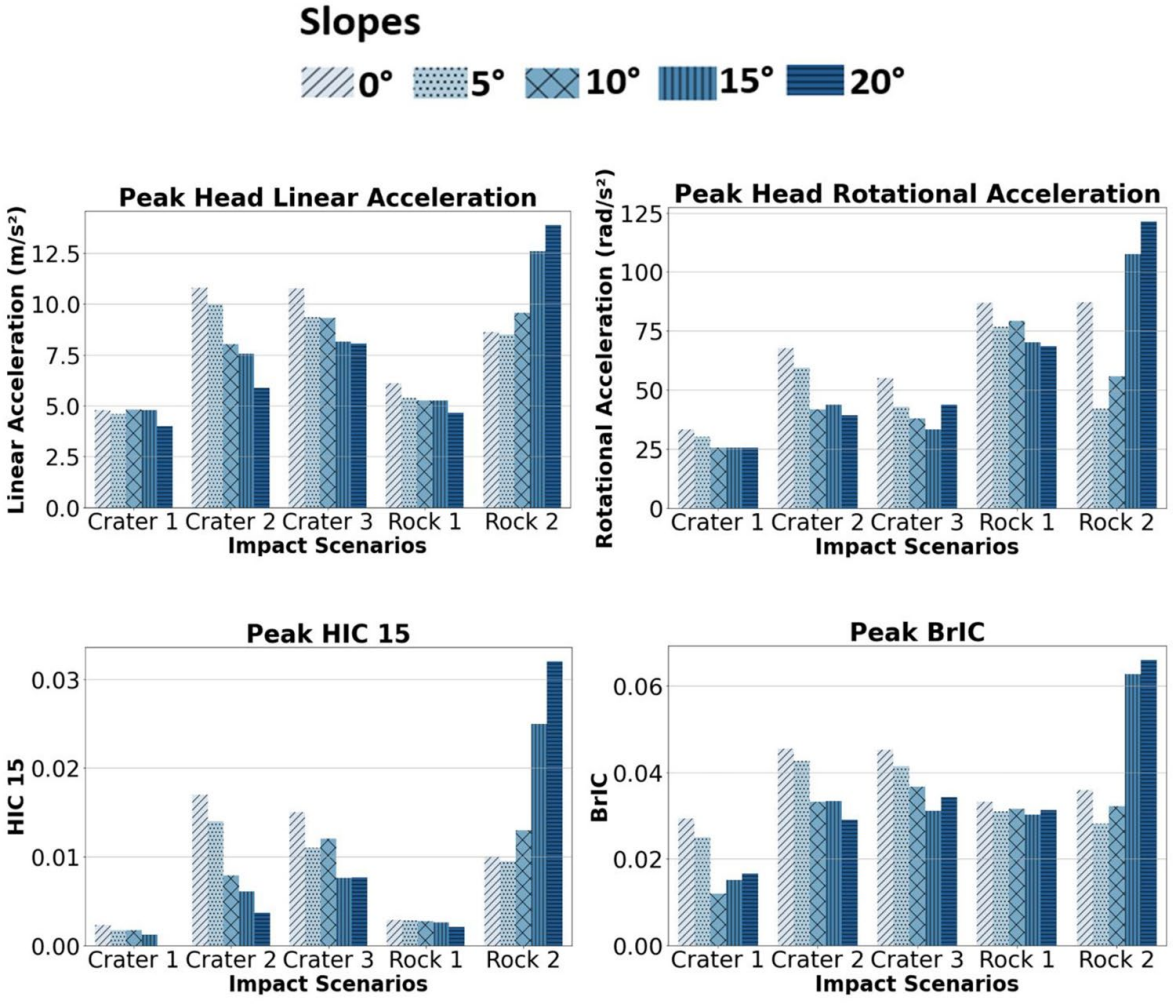
Summary of the peak head linear and rotational accelerations, HIC15 and BrIC values of 3 craters and 2 rocks over 5 slope inclinations

All head injury metric peaks are compared in Fig. 3. The most elevated peaks of head injury metrics were observed in the rock 2 scenario on a 20° slope. In the case of craters 2 and 3, the peak magnitudes of head injury metrics decreased with increasing slope inclinations. However, for crater 1 and rock 1 scenarios, the influence of slope inclination on peak magnitudes was minimal. Importantly, all scenarios remained below NASA’s injury risk threshold specified by the IARV for head injury metrics (Head linear acceleration IARV: 10 G, head rotational acceleration IARV: 2200 rad/s^2^, HIC_15_ IARV: 340 and BrIC IARV: 0.12). All peaks in the response curves were observed, with exemplary response curves for head linear acceleration for crater 2 included in Supplementary Material, Fig. 2.

#### Neck and Lumbar Spine

**Fig. 4:**
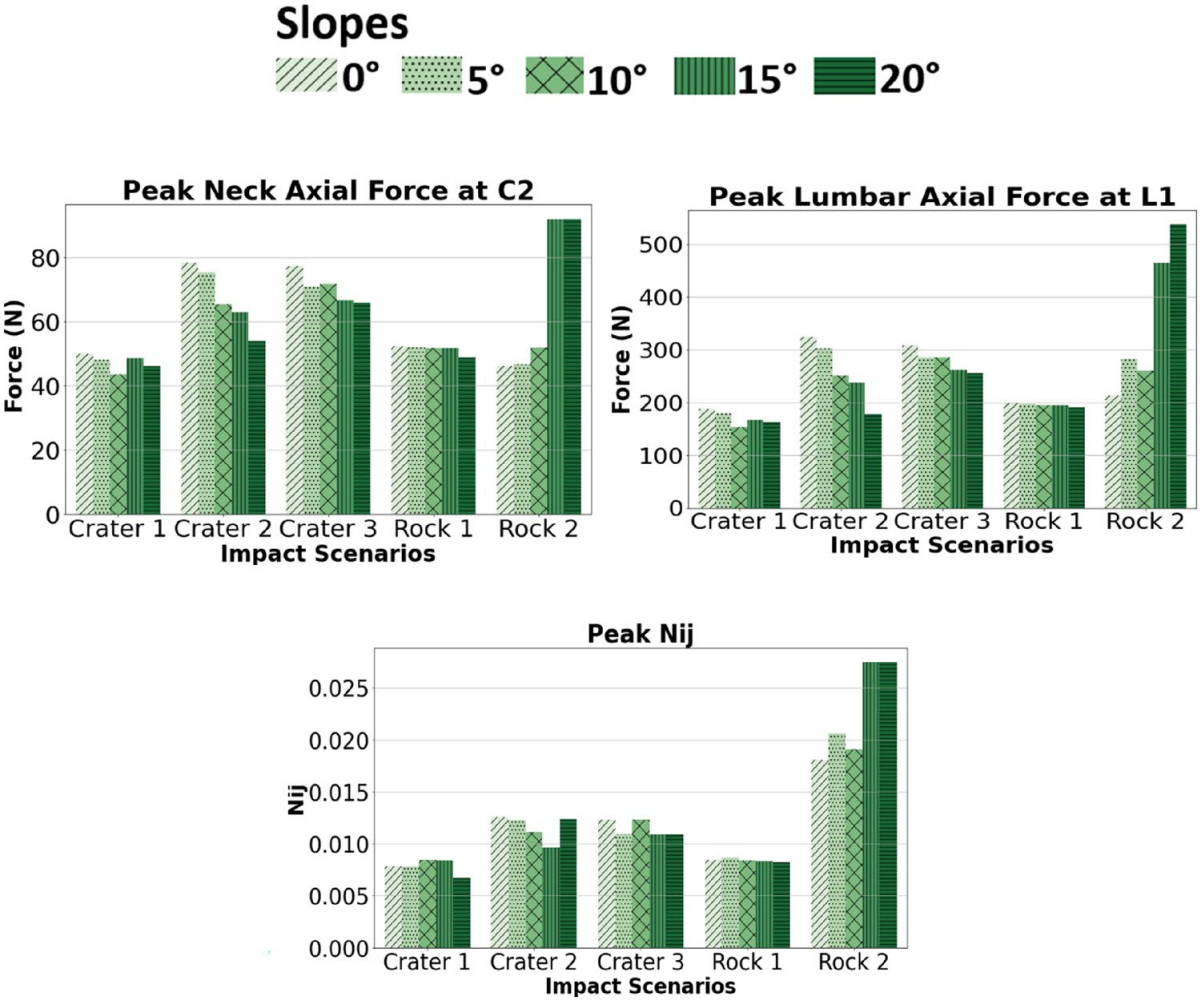
Comparison of the peak neck axial forces at the C2 vertebral level, peak lumbar axial forces at the L1 vertebral level and neck injury criterion (Nij) for all impact scenarios (3 craters and 2 rocks) over 5 slope inclinations

Similarly, all neck and lumbar peak injury metrics in Fig. 4 were below NASA’s IARV tolerance thresholds (Neck compressive force IARV: 1100 N, *N*_*ij*_ IARV: 0.16, and lumbar force IARV: 5300 N). Rock 2 had the highest neck and lumbar peak injury metrics. A notable tendency is seen on the rock 2 scenario where injury metric peaks increase as the slope inclinations increase. Injury metric peaks for crater 1 and rock 1 were unaffected by increase in slopes. All peaks in the response curves were observed, with exemplary response curves for lumbar axial force for crater 2 included in Supplementary Material, Fig. 3.

#### Lower Extremities

**Fig. 5:**
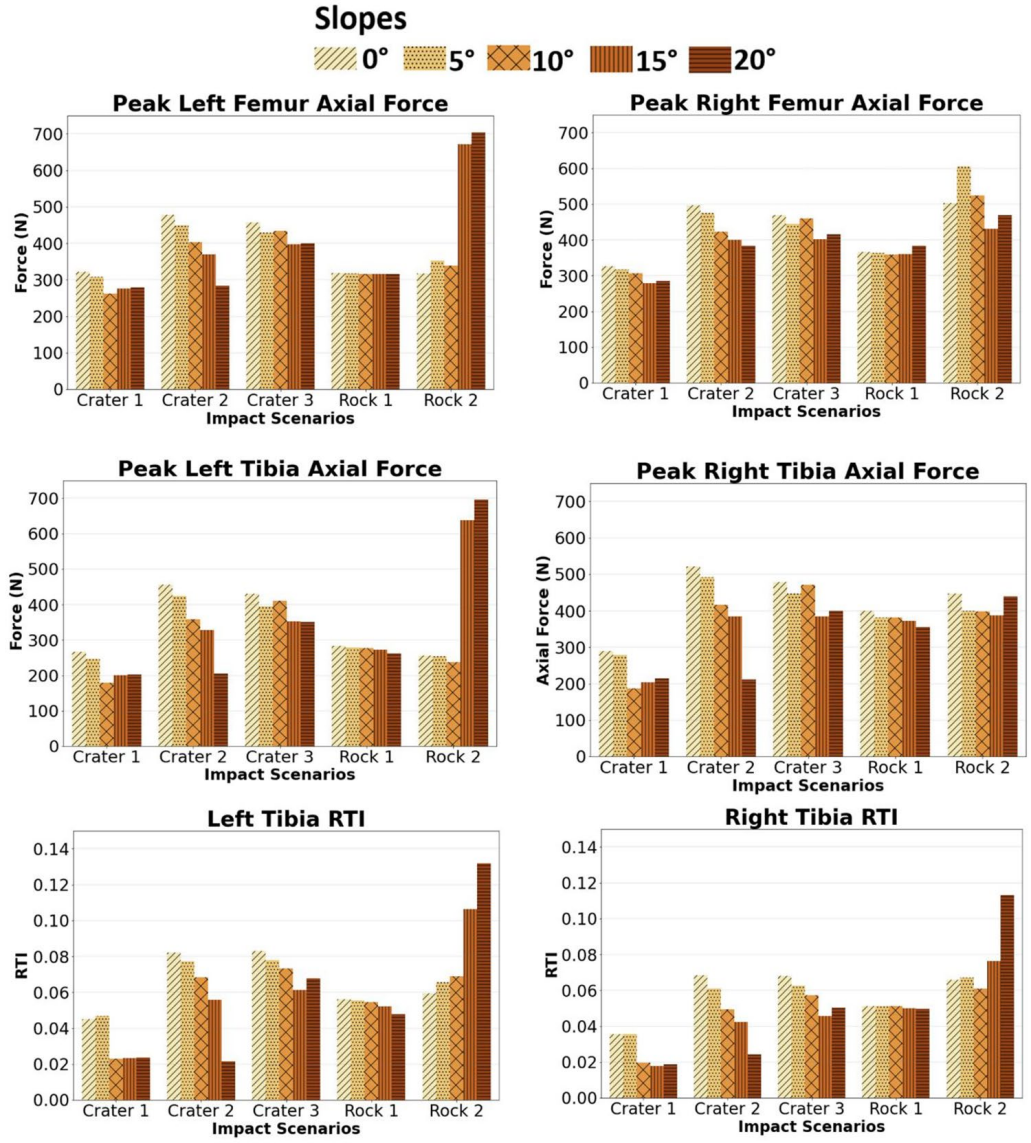
Comparison of the peak femur and tibia axial forces, and revised tibia index (RTI) values for all impact scenarios (3 craters and 2 rocks) over 5 slope inclinations

Across all loading impact scenarios, the peak injury metrics for the lower extremities remained below NASA’s tolerance limit set by the IARV (Femur compressive force IARV: 2400 N, tibia compression force IARV: 1350 N, and RTI IARV: 0.43). The highest magnitude of femur axial force was observed in the left femur of the rock 2 scenario with a 20° slope, while the highest magnitude of right femur axial force was observed in the rock 2 scenario with a 5° slope (Fig. 5). Notably, for the crater 2 impact scenario, the peak injury metric values for the lower extremities decreased as the slope inclinations increased. However, changes in slope inclinations had no significant effect on the rock 1 impact scenario. Similarly, changes in slope inclinations did not affect left or right RTI peaks for rock 1. On the other hand, RTI peaks remained highest on the rock 2 scenarios. All peaks in the response curves were observed, with exemplary response curves for femur and tibia axial force for crater 2 included in Supplementary Material, Figs. 4 and 5.

#### Body Motion Envelopes

**Fig. 6:**
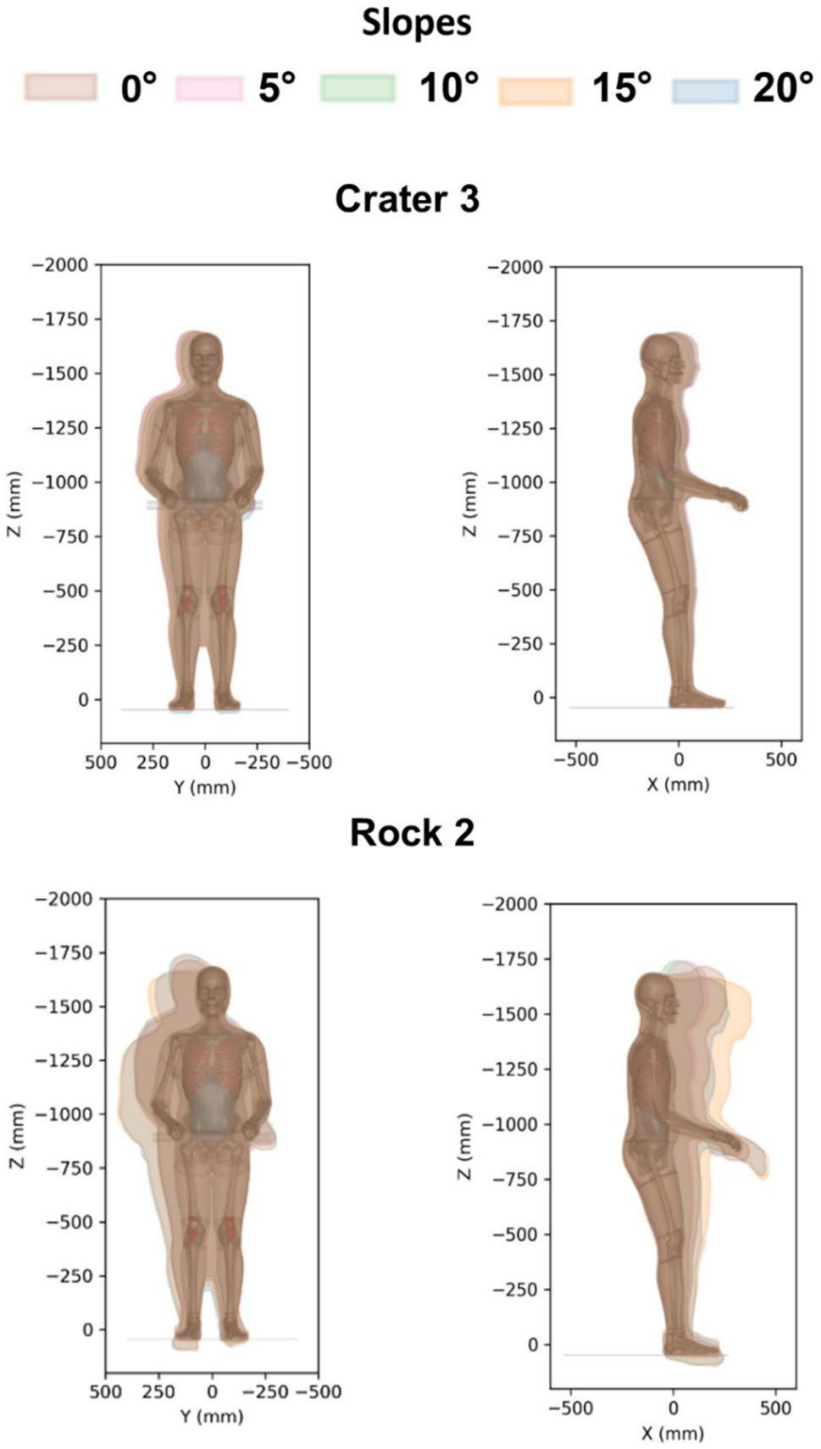
Body motion envelopes for all crater 3 and rock 2 lunar transit scenarios

Astronaut body motion envelopes for all impact scenarios were created (Fig. 6 and Supplementary Material: Figs. 6 and 7). Astronaut kinematic motion was mainly characterized by forward head and chest motion as well as lateral motion (chest displacement curves are provided for the rock 2 scenario in Supplementary Material: Figs. 8 and 9). In some cases, the head moved upwards or downwards.

### Statistical Analysis Results

All body injury metrics were positively correlated with LTV normalized peak-to-peak resultant linear acceleration (Table 2). For most injury metrics (10/13), the associations were statistically significant except for head rotational acceleration, peak right femur axial force and right RTI. The highest associations were found with head linear acceleration and neck axial force peaks. Additionally, head rotational acceleration was found to be significantly and positively correlated with normalized peak-to-peak LTV roll, pitch and yaw angle displacements, with roll angle having the strongest correlation.

Simple linear regression models revealed all injury metrics except head rotational acceleration are significantly associated with resultant peak-to-peak linear acceleration of the LTV (Table 3). Linear regression models revealed the highest increases were in the neck, lumbar and femur axial forces per unit increase in LTV resultant linear acceleration. The peak-to-peak LTV resultant linear accelerations explained between 11.6 and 70.6% of the observed variation in the resulting injury metrics. The maximum variation (~70.6%) contributed to resultant linear acceleration was on head linear acceleration, whereas resultant linear acceleration had little effect on head rotational acceleration and right RTI. Variation in peak axial force at the C2 vertebral level is also highly explained by LTV resultant linear acceleration. Injury metrics consistently demonstrated a linear relationship when plotted against LTV normalized resultant acceleration (Supplementary material: Figs. 10–16).

**Table 3:** Linear regressions coefficients (*β*), *R*^2^ expressed as percentages, and *p* values for all body injury metrics with resultant peak-to-peak linear accelerations as predictors

Linear regression models coefficients		$R^{2}$	*p* value	$\beta$
Injury Metrics	Head linear acceleration	**70.6**	** < 0.0001**	**0.709**
	Head rotational acceleration	1.4	0.5249	1.115
	HIC15	**40.8**	**0.0006**	**0.001**
	BrIC	34.8	0.0019	0.002
	Peak axial force @ C2	**82.9**	** < 0.0001**	**3.818**
	Nij	**11.6**	** < 0.0001**	**0.001**
	Peak axial force @ L1	**39.9**	**0.0007**	**15.446**
	Peak left femur axial force	**40.7**	**0.0006**	**19.124**
	Peak right femur axial force	31.3	0.0037	15.296
	Peak left tibia axial force	**40.5**	**0.000631**	**21.891**
	Peak right tibia axial force	**27**	**0.0007**	**14.626**
	Left RTI	**32.6**	**0.00287**	**0.004**
	Right RTI	7.9	0.173	0.002

Statistically significant regressions are bolded and underlined. Beta coefficients indicate the increase in each respective body region injury metric per m/s^2^ unit increase in peak-to-peak linear acceleration of the LTV

## Discussion

This study simulated potential unpressurized lunar rover transit scenarios that may occur during EVAs, involving traversing across craters or rocks, to assess astronaut potential risks. Various lunar transit impact scenarios were simulated, considering different lunar obstacles (craters or rocks), variations in crater depths and diameters, rock heights, and slope inclinations. Driving an unpressurized vehicle under lunar gravity can pose a significant risk of injury to the astronaut, potentially resulting in fatality. Furthermore, this study compared the injury metrics obtained with the corresponding IARVs reported in existing literature. All injury metrics were below NASA’s IARV thresholds, suggesting the feasibility of maintaining an upright posture on the LTV without exceeding the acceptable injury risk levels established by NASA.

In general terms, hitting a crater or rock is a low severity impact characterized by peak acceleration pulse magnitudes. The highest peak-to-peak acceleration magnitudes remained around 14–15 m/s^2^ (1.55 G’s). These values are comparable with Wang’s study, which reported rover body accelerations ranging from approximately 5 m/s^2^ to 14 m/s^2^ for deformable and rigid roads, respectively [7]. It is important to note that various rover model parameters, such as wheel dimensions, suspension characteristics, impact object dimensions, and rover velocity, can also influence acceleration peaks. Impacts on craters over small slope inclinations (0°–5°) had smaller acceleration magnitudes. Rotational displacements generally remained within a lower range for most crater scenarios, with the highest displacement at approximately 3°. On the other hand, rock impact scenarios resulted in more severe rotational displacements, ranging from 2° up to 7°. Additionally, the acceleration pulses obtained from TRICK simulations indicated that impacts with craters or rocks on the Moon typically involve multi-spike acceleration profiles. To accommodate these dynamic characteristics within the simulation, the impacts were divided into two major events (events 1 and events 2). These spikes represent different wheel interactions with lunar soil including the front wheel falling into the crater or hitting the bottom of the crater. Event 1 generally exhibited greater severity across most scenarios, with some exceptions observed in craters 1 and 2, as well as rock 2, where the second event demonstrated greater severity.

Across all 25 impact scenarios, all body injury metrics remained below NASA’s IARV tolerance limits. Notably, HIC_15_ values remained consistently low, measuring below a tenth of a unit. On the other hand, highest rotational acceleration peaks typically occurred at lower slope inclinations across most scenarios, except for rock 2. No direct acceleration pulse was transmitted to the astronaut’s head via the vehicle, contributing to the overall low peak head injury metrics observed. Differences in peak rotational accelerations (and BrIC) across various slopes stem from variations in the magnitude of the prescribed linear accelerations governing the LTV motions. Notably, these injury metrics have higher values in lower slopes likely due to the suspension dynamics when encountering obstacles at inclined angles. The force exerted by suspension springs is contingent upon the degree of compression they undergo, which can alter across the different slopes. Also, the vehicle inclination varies the force components of the vehicle dynamics. Consequently, linear accelerations may decrease with increasing slope inclination. For rock 2, there is a tendency for injury metrics to rise again on the two steepest inclines. It is important to highlight that in these two cases, the modeled rover in the TRICK simulations encountered difficulties overcoming the rock obstacle, potentially leading to heightened acceleration pulse magnitudes on 15 and 20-degree slopes. Moreover, differences in head rotational acceleration and BrIC are consistently of small magnitude, indicating minimal changes in head injury risk among the various scenarios considered in the study. Lumbar and neck injury metrics revealed low magnitude compression forces. Lower body axial forces, including femur and tibia, showed magnitudes similar to those observed for the lumbar region. Axial forces observed in the lumbar region were higher than those in the neck, aligning with the expected load transmission pattern from the lower and trunk regions of the body to the neck and head. This suggests that the lower extremities and trunk regions are most susceptible to injury if LTV accelerations increased.

A notable trend was observed in the craters, where axial forces in the lower extremities decreased as slope inclinations increased. This trend can also be attributed to the decrease in acceleration pulse magnitude, as the astronaut’s initial position and conditions remained unchanged. In the rock 2 scenarios, higher peak compression forces were observed in the left femurs and tibias compared to the right counterparts. This difference can be attributed to the dynamics and motion of the floor panel resulting from the impact on only one wheel of the LTV. To evaluate the risk of fracture, RTI values were calculated for all scenarios, and all values remained below the unity value. None of these values exceeded NASA’s IARV tolerance limit of 0.43 for RTI. Peak force values for the rock 1 scenarios were unaffected by changes in slope inclinations. It is important to note that LTV acceleration magnitudes remained within a similar range for the rock 1 scenario across all slope inclinations.

Findings from this study may have applicability to other fields, such as micro-mobility transportation (scooters, two-wheel Segway human transporters, all-terrain vehicles/four-wheelers, etc.) and mass transportation. Understanding the primary body regions most affected by road transient inputs, particularly the lumbar and lower extremities, is essential for enhancing safety measures within these fields. For instance, in the urban bus study by Palacio et al., the knees were found to have a higher probability of injury compared to the head [9]. Our findings align with these results, as evidenced by higher injury metrics and axial forces observed in the lower extremities relative to other body regions. Additionally, consistent low HIC_15_ values observed in the study by Palacio et al., except for one case, are mirrored by our results [9]. It’s important to note the potential variability in HIC values when studying head-spacesuit interactions. Furthermore, comparing tibia injury metrics between mass transport studies and our research, it is evident that RTI remained higher in the former, with a range of 0.12 to 1.2 compared to our results ranging from 0.02 to 0.13 [9]. It’s worth highlighting that lunar gravity could influence both the temporal and magnitude effects in acceleration pulses, along with body injury metrics.

Furthermore, body kinematic responses were assessed through the development of body motion envelopes for all simulated scenarios. These envelopes provided quantifications of the excursion of unrestrained body regions, aiming to inform future spaceflight and LTV designs. Our results revealed that the motion of the head is primarily forward, with the highest forward motion observed in crater 2 among the crater scenarios and in rock 2 among the rock scenarios. The maximum forward motion of the head was approximately 375 mm. Similarly, the chest exhibited a predominantly forward motion, with the highest excursion measuring 260 mm. These head and chest excursions will be crucial considerations to prevent impacts between the head, collarbone, and chest with spacesuits, helmets, or the LTV. Additionally, lateral head impacts pose a risk, as the range of lateral arm and head motions varied from 65 to 240 mm. Moreover, upward and downward head motions were observed in the motion envelopes, particularly in the rock 2 scenarios, with excursions reaching approximately 85 mm.

Significant associations were observed between most injury metrics and the resultant peak-to-peak linear acceleration of the LTV. Furthermore, head rotational acceleration was significantly correlated with all rotational angle displacements (roll, pitch, and yaw). Additionally, the rotational angles demonstrated significant correlations with the right RTI, a metric accounting for tibia bending moment. Injury metrics consistently exhibited linearity against LTV normalized resultant acceleration. Nevertheless, two outliers were observed in the lumbar, left femur and left tibia axial forces. These two data points specifically relate to rock 2, slopes of 15° and 20°, where the rover encountered difficulty overcoming obstacles in the TRICK simulations, which prompted to the utilization of the parametric Kendall-Theil Sen Siegel method for regression models. Our regression models revealed that variations in head acceleration-based injury metrics, neck, lumbar, and lower extremity axial forces can be attributed to the magnitude of the acceleration pulse. However, other factors may also contribute to these variations.

In this study, a total of 25 simulations were conducted encompassing 5 impact scenarios with variations in slope inclinations, as well as dimensions of craters and rocks. One limitation of the study is the relatively small sample size, and a larger number of simulations for each type of scenario would enhance robustness. Given that the LTV to be used in upcoming Artemis missions is currently in the design development phase, there is limited information available regarding restraint systems and the structural components of the LTV. To address this limitation, we utilized simplified buck and restraining systems in our modeling approach. Incorporating parameter variations in both the length and placement of the foot straps could be considered in future studies. It is important to note that a rover overcoming a crater or rock can have a duration of up to 3 s. While explicit computational models provide valuable information regarding peak injury metrics for severe impact regions, it’s plausible that astronaut positioning may undergo changes and not fully recover to a nominal position after event 1 (Supplementary information: Figs. 8 and 9). Hence, for future investigations, it would be pertinent to either simulate the entire pulse or each event sequentially. Additionally, the simulations were conducted under the assumption that the astronaut is in a relaxed state, responding to perturbations to restore their posture to its initial state. Should the astronaut choose to brace, these models have the capability to simulate such conditions by specifying muscle activation values at the beginning of the simulation [18, 29]. However, data on muscle activation for all muscles are scarce. Moreover, future studies ought to account for the kinematic response of astronauts while wearing spacesuits, as well as the alterations in lower extremity or lumbar compression loads. The additional weight from spacesuits can impact injury metrics, potentially leading to injury risk thresholds being exceeded. Finally, the posture of the GHBMC model might slightly vary during simulations when rotated to a target inclination and should be considered in the future.

In conclusion, hitting a crater or a rock is a low severity impact characterized by multiple acceleration spikes. Even though astronauts have driven a rover in a seated posture, this study assessed body kinematics and injury risks when manipulating a rover in an upright posture. All body injury metrics were under NASA’s tolerance thresholds highlighting the low risk for body injuries. Injury metrics decreased along the load path, from the lower body (highest metrics) to the upper body (lowest metrics). This study also considers lunar gravity-induced temporal effects on transient acceleration pulses as well as the effects of rotational displacement on the injury metrics and kinematic response. While injury metrics were low, increased upper body motion could potentially pose a risk of injury from flail and occupant interaction with the surrounding vehicle, suit, and restraint hardware. The data generated will also serve for comparison with other postures on the LTV and aid in the design phase of the EVA spacesuit and LTV.

### Supplementary Information

Below is the link to the electronic supplementary material.

Supplementary file1 (PDF 1422 KB)
