# Supplementary material for: Injury Risk Predictions in Lunar Terrain Vehicle (LTV) Extravehicular Activities (EVAs): A Pilot Study
Source: Ann Biomed Eng. 2024 Jun 5;52(9):2534–45. doi: 10.1007/s10439-024-03543-8 (PMC11329544; doi:10.1007/s10439-024-03543-8)
Supplement: Supplementary file 1 — Supplementary file1 (PDF 1422 KB) [file 10439_2024_3543_MOESM1_ESM.pdf]

Supplementary Information

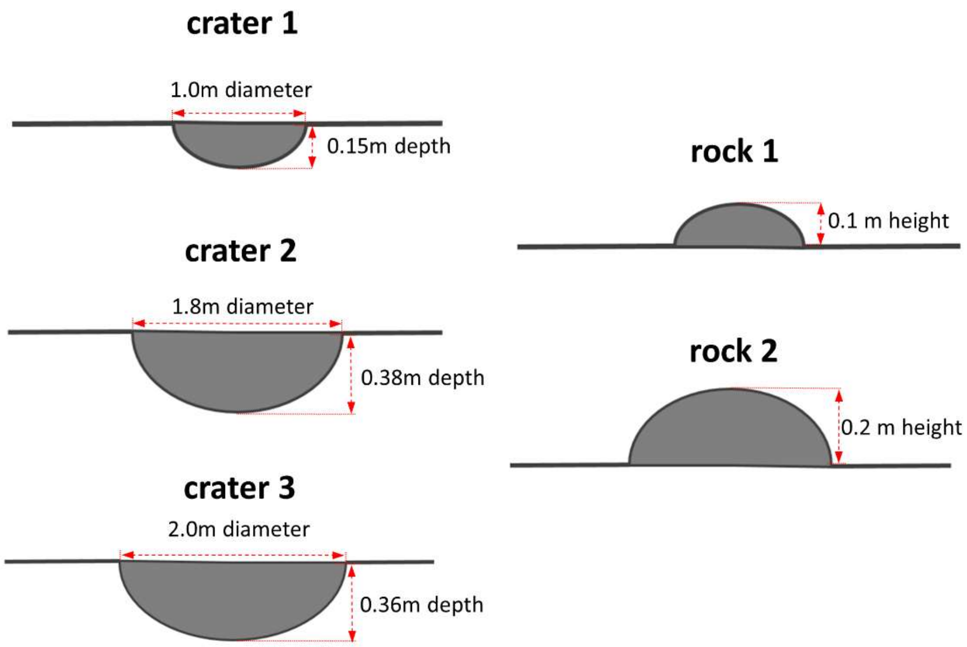

Fig. 1 Lunar surface obstacles (craters and rocks) dimensions

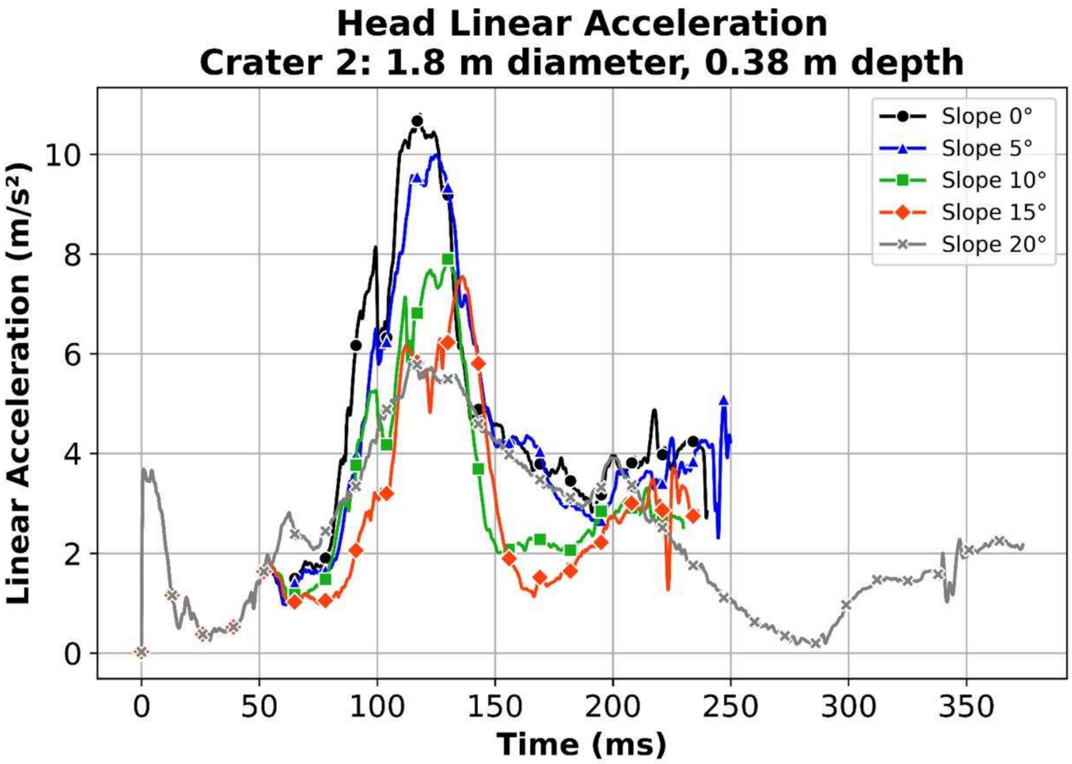

Fig. 2 Head linear acceleration response curves for crater 2 scenario (0, 5, 10, 15, and 20 degrees slopes)

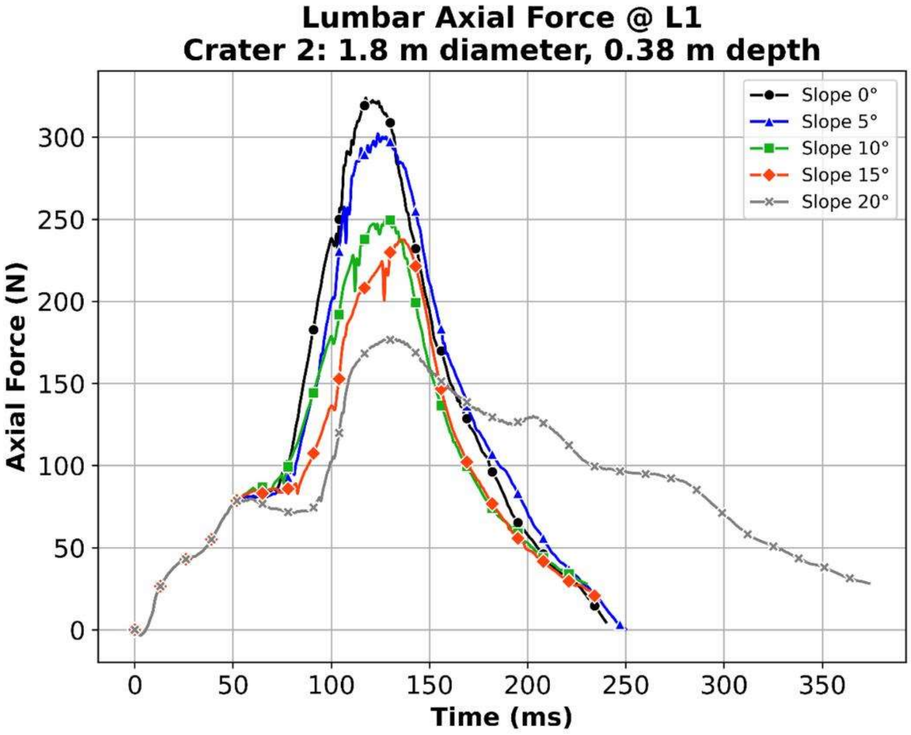

Fig. 3 Lumbar axial force at L1 vertebral level response curves for crater 2 scenario (0, 5, 10, 15, and 20 degrees slopes)

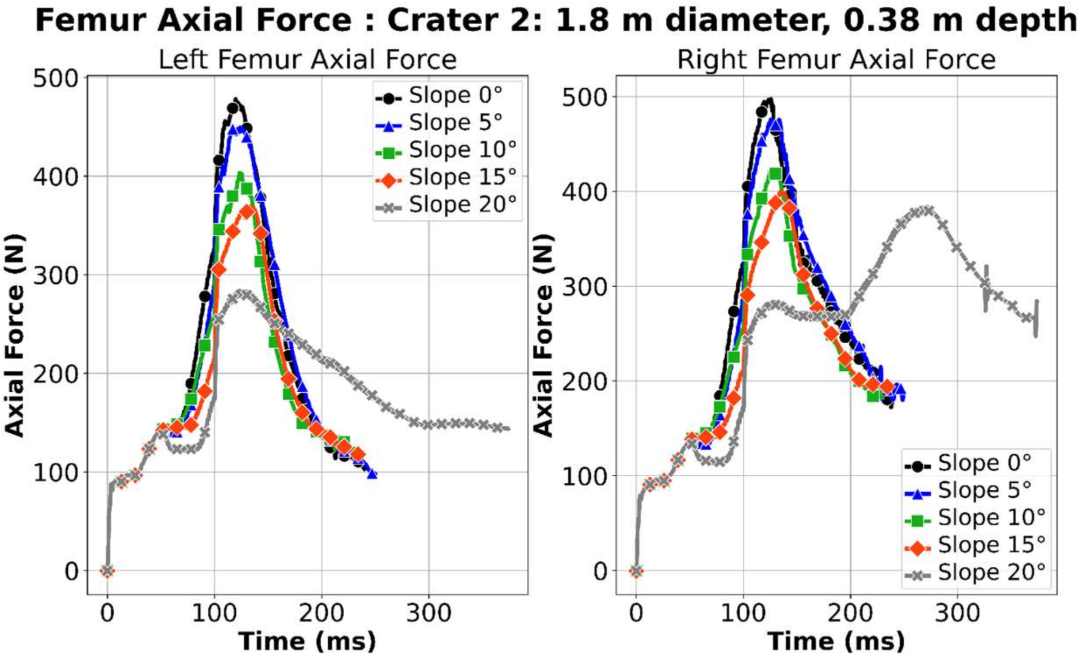

Fig. 4 Left and right femur axial force response curves for crater 2 scenario (0, 5, 10, 15, and 20 degrees slopes)

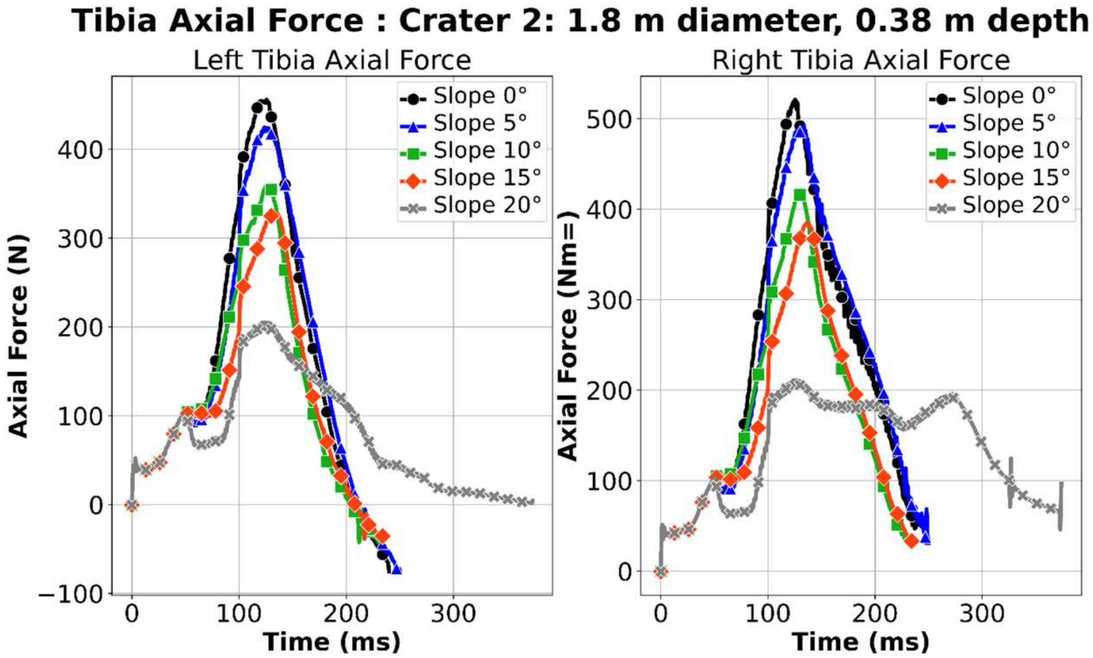

Fig. 5 Left and right femur axial force response curves for crater 2 scenario (0, 5, 10, 15, and 20 degrees slopes)

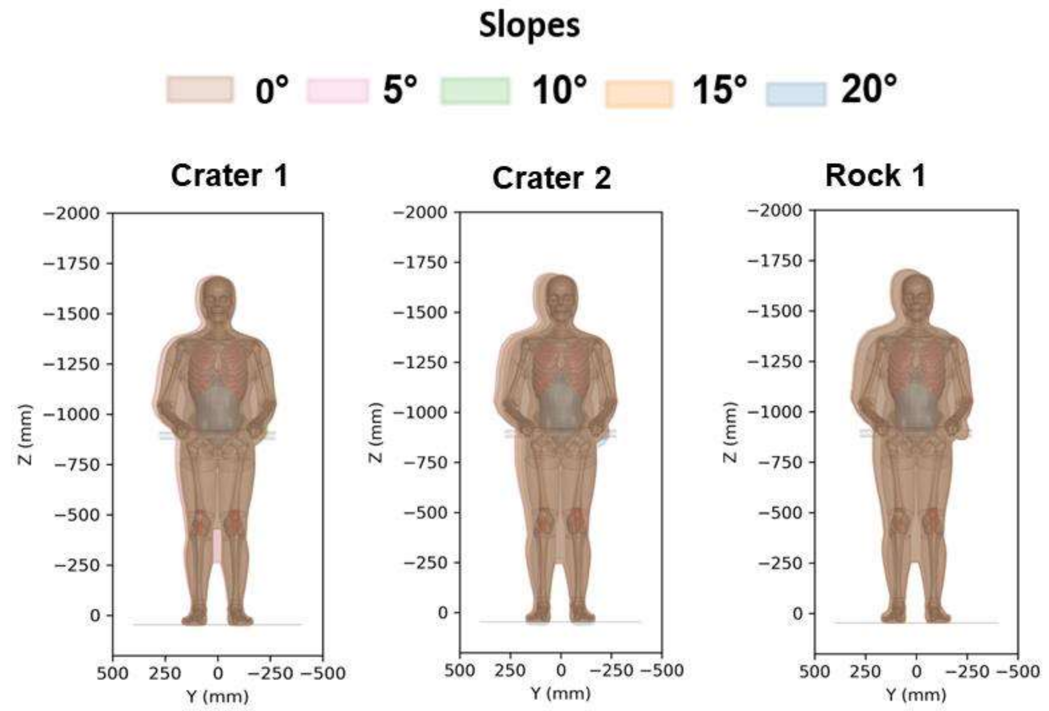

Fig. 6 Front View Body Motion Envelopes for all crater 1, crater 2 and rock 1 lunar transit scenarios.

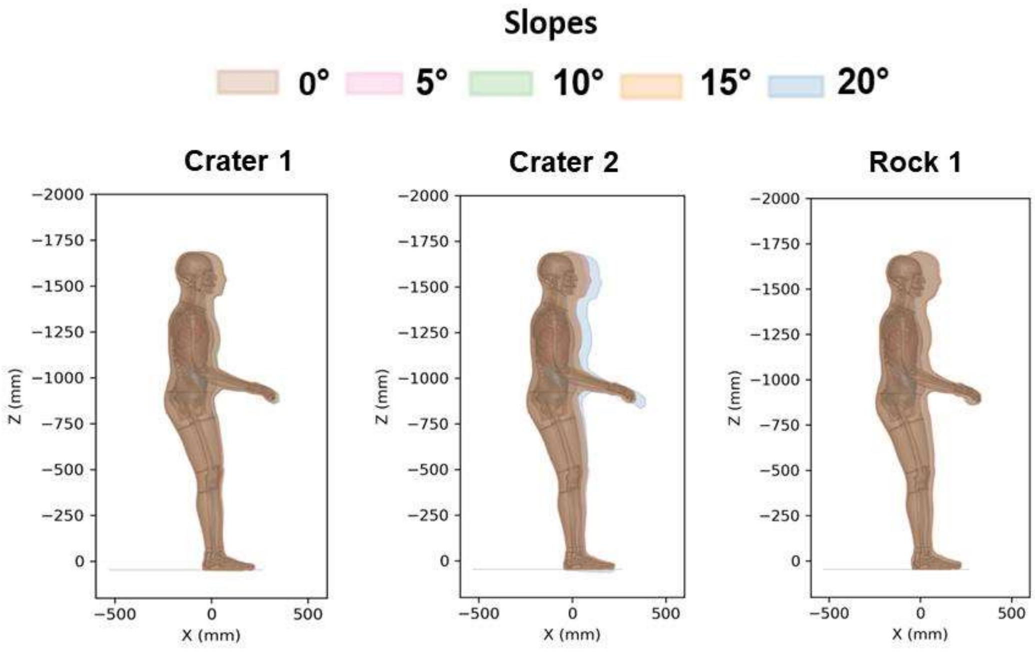

Fig. 7 Side View Body Motion Envelopes for all crater 1, crater 2 and rock 1 lunar transit scenarios.

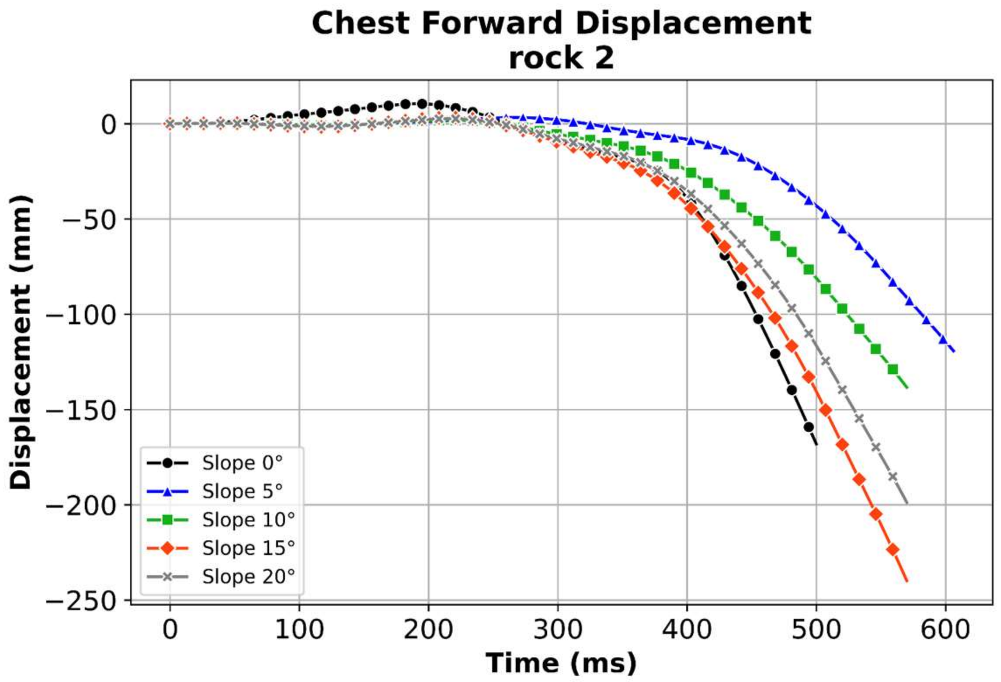

Fig. 8 Chest forward displacement response curves for rock 2 scenario (0, 5, 10, 15, and 20 degrees slopes)

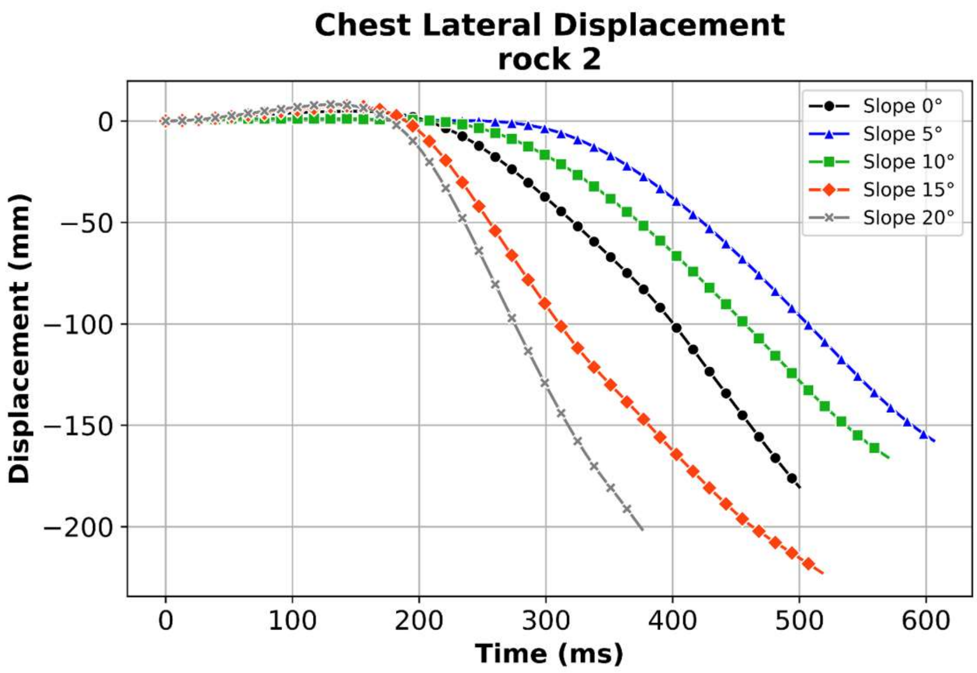

Fig. 9 Chest forward displacement response curves for rock 2 scenario (0, 5, 10, 15, and 20 degrees slopes)

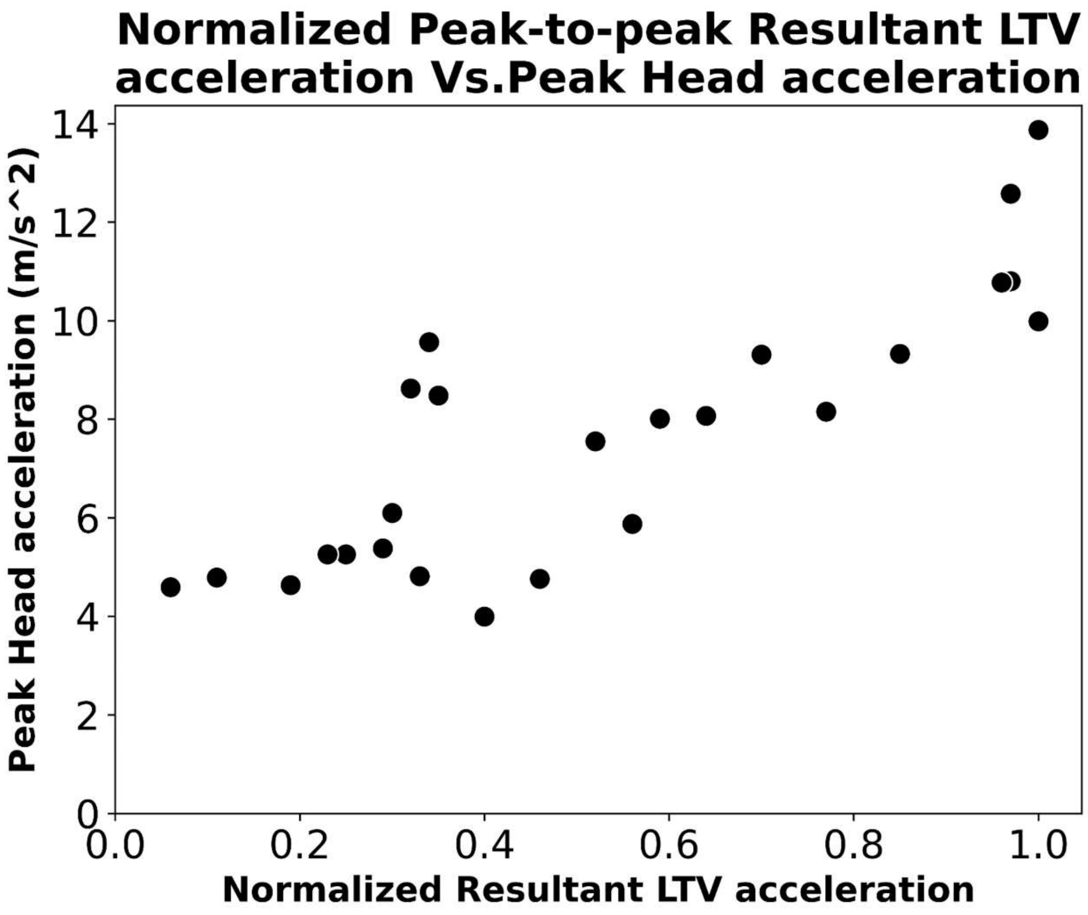

Fig. 10 Scatterplot of normalized peak-to-peak resultant LTV linear acceleration Vs. peak head linear acceleration

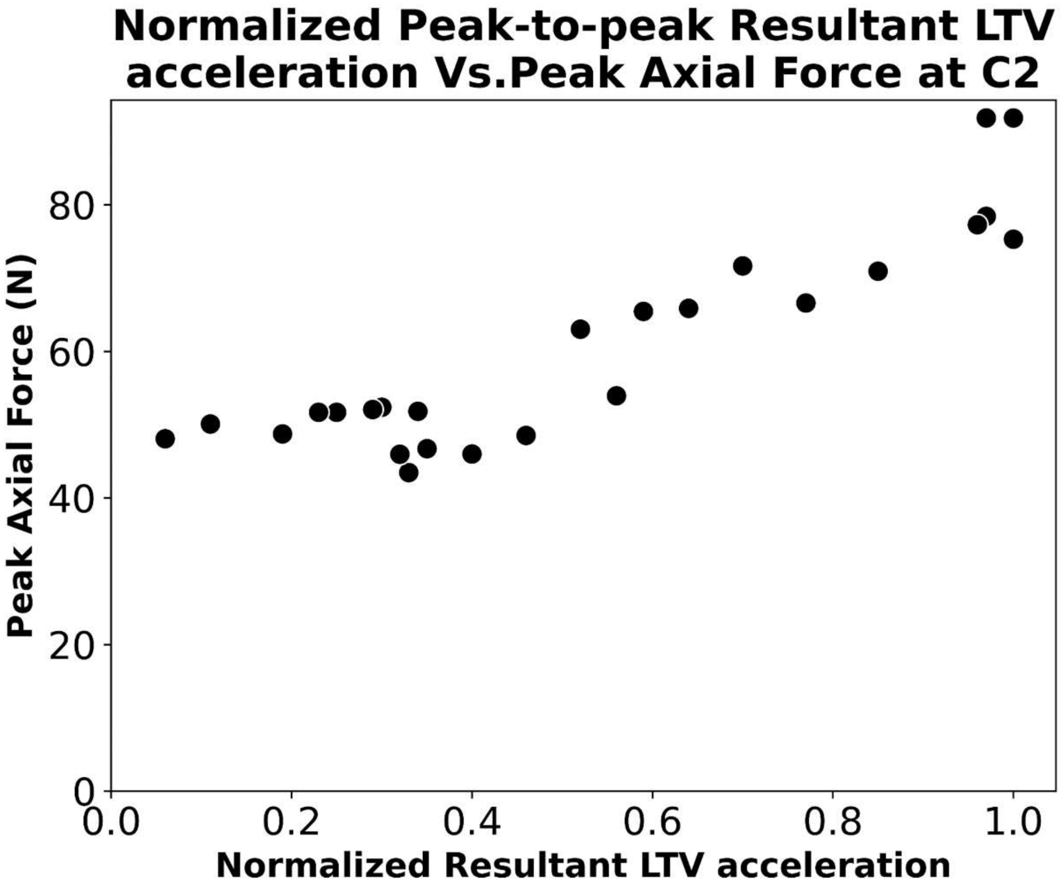

Fig. 11 Scatterplot of normalized peak-to-peak resultant LTV linear acceleration Vs. peak axial force at C2 vertebral level

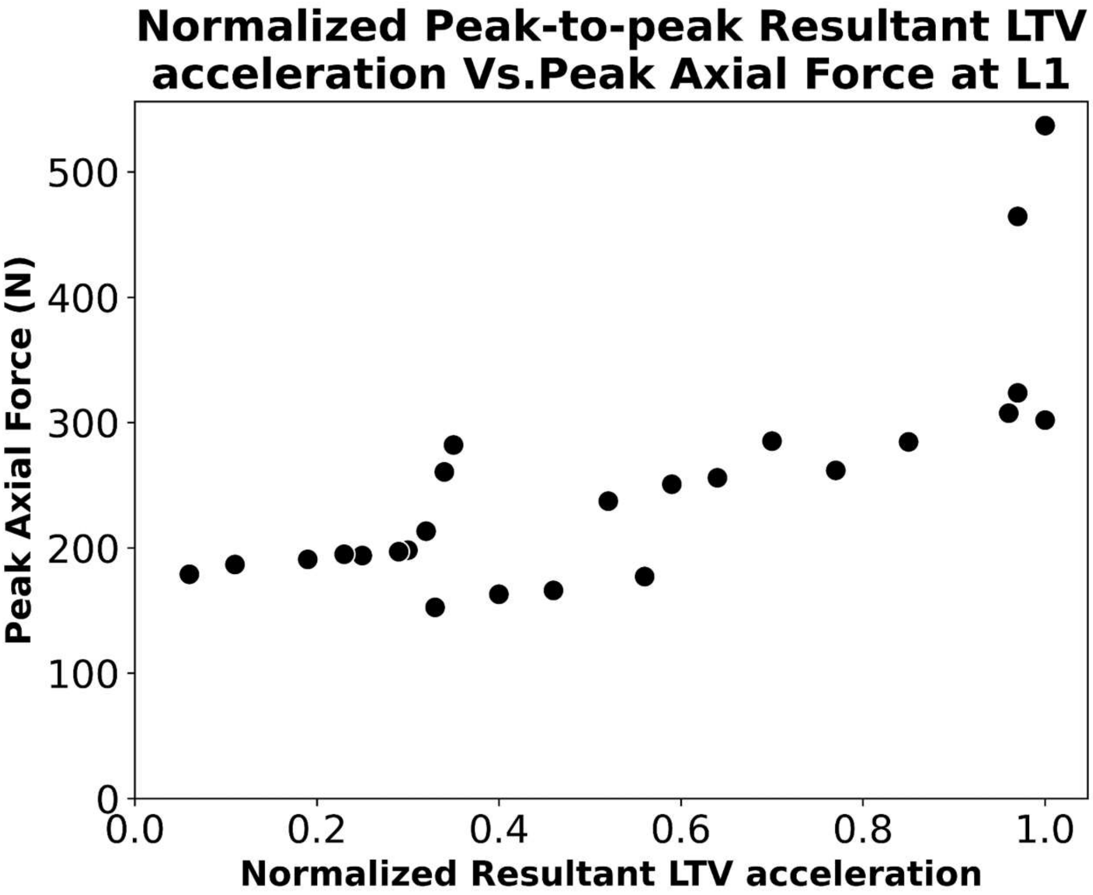

Fig. 12 Scatterplot of normalized peak-to-peak resultant LTV linear acceleration Vs. peak axial force at L1 vertebral level

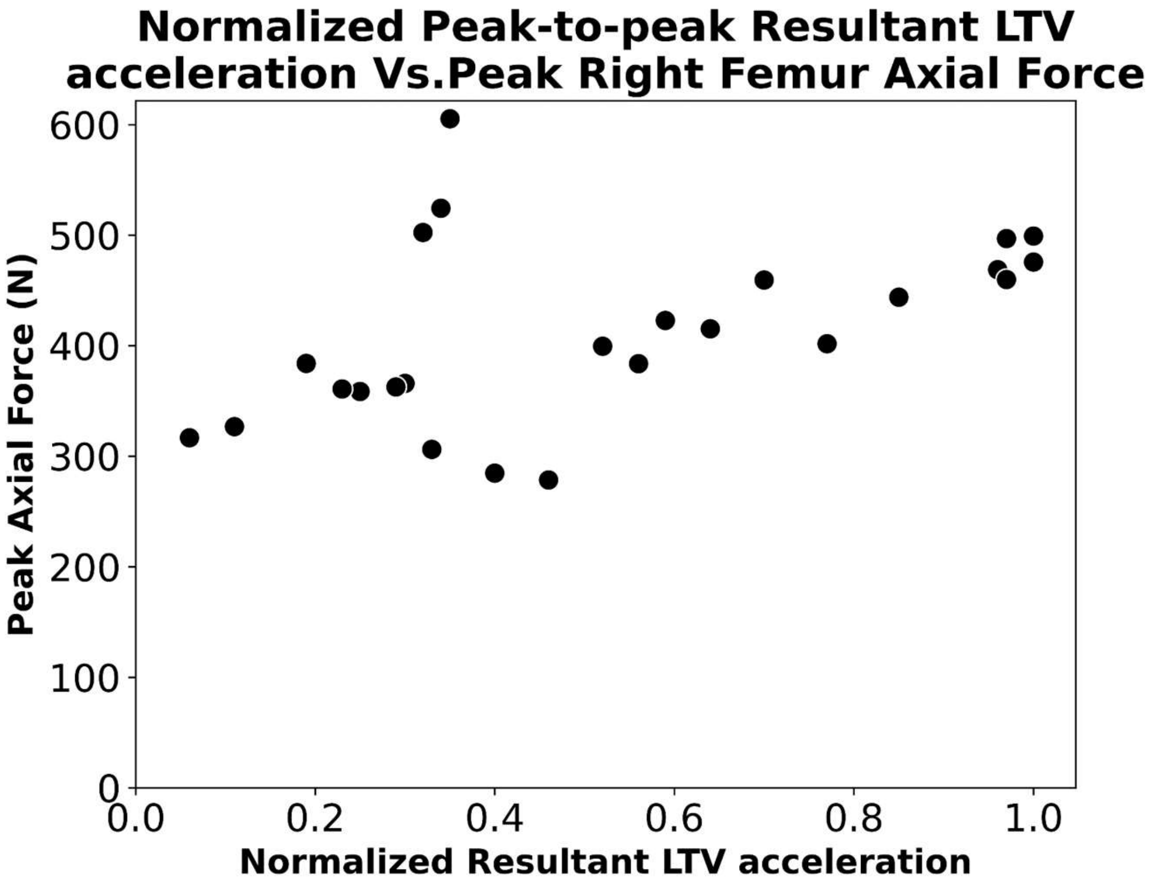

Fig. 13 Scatterplot of normalized peak-to-peak resultant LTV linear acceleration Vs. peak right femur axial force

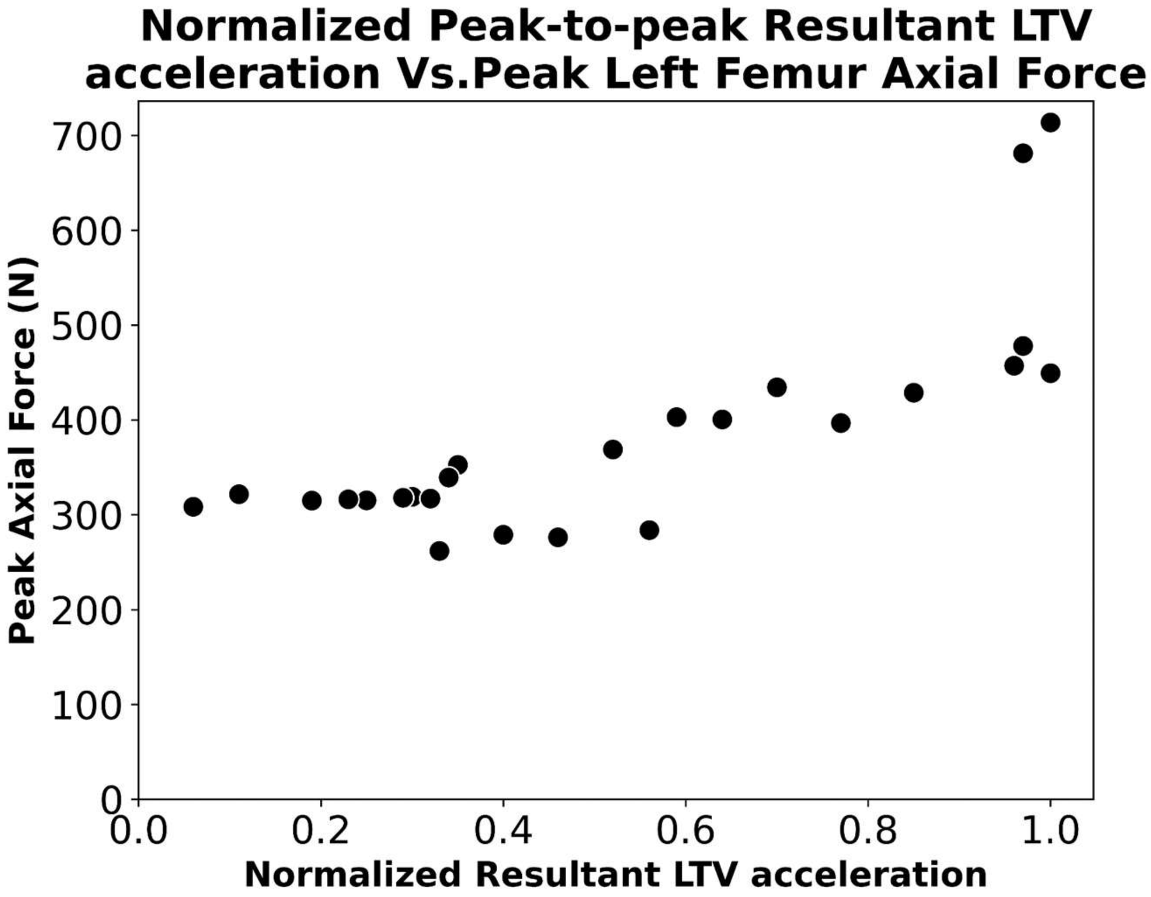

Fig. 14 Scatterplot of normalized peak-to-peak resultant LTV linear acceleration Vs. peak left femur axial force

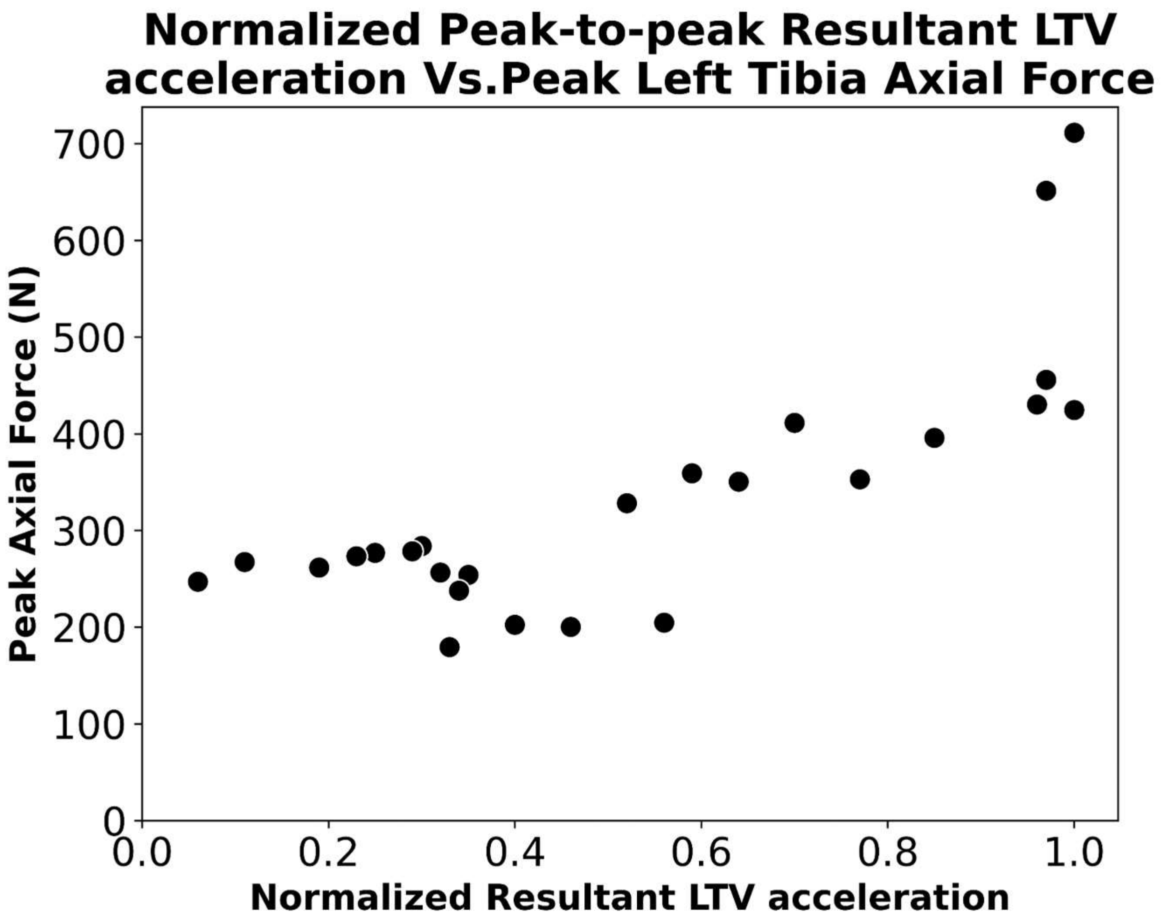

Fig. 15 Scatterplot of normalized peak-to-peak resultant LTV linear acceleration Vs. peak left tibia axial force

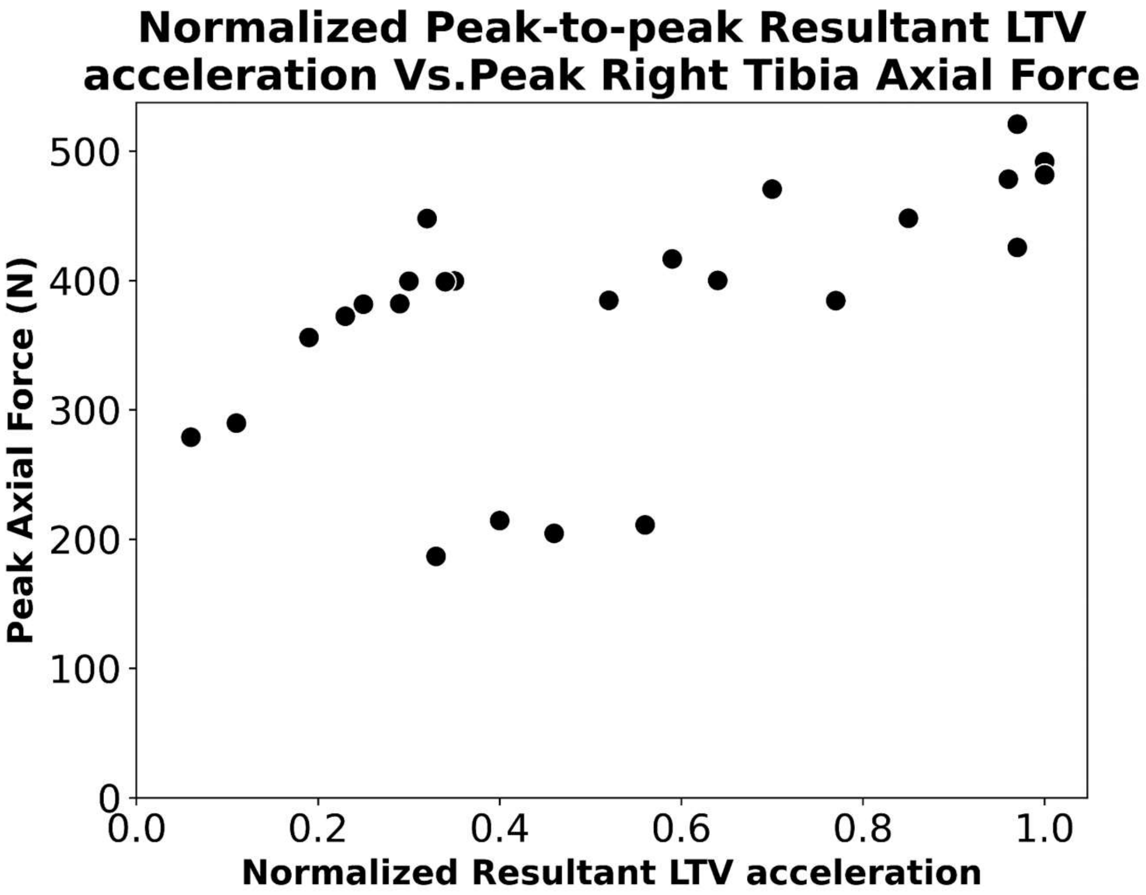

Fig. 16 Scatterplot of normalized peak-to-peak resultant LTV linear acceleration Vs. peak right tibia axial force
